# Novel functions for 2‐phenylbenzimidazole‐5‐sulphonic acid: Inhibition of ovarian cancer cell responses and tumour angiogenesis

**DOI:** 10.1111/jcmm.14989

**Published:** 2020-01-20

**Authors:** Min Su Kim, Jae Hyeon Kim, Eun‐Kyung Ahn, Young‐Rak Cho, Surim Han, Choong‐Hyun Lee, Gyu‐Un Bae, Joa Sub Oh, Kyu‐Bong Kim, Dong‐Wan Seo

**Affiliations:** ^1^ Department of Pharmacy College of Pharmacy Dankook University Cheonan Korea; ^2^ Biocenter Gyeonggi Business & Science Accelerator Suwon Korea; ^3^ Department of Chemistry College of Natural Science Dankook University Cheonan Korea; ^4^ Department of Pharmacy College of Pharmacy Sookmyung Women’s University Seoul Korea

**Keywords:** 2‐phenylbenzimidazole‐5‐sulphonic acid, angiogenesis, ovarian cancer, p38^MAPK^

## Abstract

In this study, we investigated the effects and molecular mechanisms of 2‐phenylbenzimidazole‐5‐sulphonic acid (PBSA), an ultraviolet B protecting agent used in sunscreen lotions and moisturizers, on ovarian cancer cell responses and tumour angiogenesis. PBSA treatment markedly blocked mitogen‐induced invasion through down‐regulation of matrix metalloproteinase (MMP) expression and activity in ovarian cancer SKOV‐3 cells. In addition, PBSA inhibited mitogen‐induced cell proliferation by suppression of cyclin‐dependent kinases (Cdks), but not cyclins, leading to pRb hypophosphorylation and G_1_ phase cell cycle arrest. These anti‐cancer activities of PBSA in ovarian cancer cell invasion and proliferation were mediated by the inhibition of mitogen‐activated protein kinase kinase 3/6‐p38 mitogen‐activated protein kinase (MKK3/6‐p38^MAPK^) activity and subsequent down‐regulation of MMP‐2, MMP‐9, Cdk4, Cdk2 and integrin β1, as evidenced by treatment with p38^MAPK^ inhibitor SB203580. Furthermore, PBSA suppressed the expression and secretion of vascular endothelial growth factor in SKOV‐3 cells, leading to inhibition of capillary‐like tubular structures in vitro and angiogenic sprouting ex vivo. Taken together, our results demonstrate the pharmacological effects and molecular targets of PBSA on modulating ovarian cancer cell responses and tumour angiogenesis, and suggest further evaluation and development of PBSA as a promising chemotherapeutic agent for the treatment of ovarian cancer.

## 1 INTRODUCTION

Ovarian cancer is the second leading cause of mortality among the gynaecologic cancers.^1^ High mortality rates of ovarian cancer patients may be due in part to a lack of highly sensitive detection methods or effective therapeutics against metastatic and recurrent phenotypes.^2^ Dysregulated activation or overexpression of cell signalling‐related molecules including receptor tyrosine kinases (RTKs) and integrins mediates aberrant activation of their downstream signalling pathways that triggers uncontrolled and imbalanced responses, suggesting the rational strategy and pharmacological efficacy of RTK/integrin‐targeted therapeutics for the treatment of ovarian cancer.^3^, ^4^, ^5^ In addition, matrix metalloproteinases (MMPs), which degrade extracellular matrix (ECM) and cellular components in the tissue microenvironment, are closely associated with cancer cell growth and progression as well as normal tissue remodelling.^6^, ^7^ High expression and activity of MMPs have been reported to contribute to aggressive phenotypes and poor survival of ovarian cancer.^8^, ^9^ However, inhibition of MMP activity without sufficient knowledge about substrate specificity or physiological roles in cancer biology has been disappointing in various cancer clinical trials.^10^ Therefore, intensive investigations of cellular and molecular networks underlying the progression of ovarian cancer may provide insights into effective therapeutic targets and strategies for the prevention and treatment of cancer.

2‐Phenylbenzimidazole‐5‐sulphonic acid (PBSA) has widely been used as a sunscreen agent to protect skin from ultraviolet (UV) radiation, resulting in reduction of sunburn, early skin ageing and skin cancer. In parallel to the protective effect against UV‐induced cyclobutane pyrimidine dimers, PBSA has also been reported to generate a variety of free radicals and exhibit photosensitizing activity, raising the possibility of phototoxic damage to cellular components including DNA, proteins and lipids.^11^, ^12^, ^13^, ^14^ Although the potential side effects and risks of PBSA to ecosystem as well as human health have been proposed in several safety and toxicity studies, chronic exposure to PBSA did not significantly induce pathological changes in the liver or gonads of rainbow trout, a common toxicity model organism.^15^ Furthermore, the phototoxic potential of PBSA could be reduced by complexation with hydroxypropyl‐β‐cyclodextrin.^16^ However, the effects and molecular mechanisms of PBSA on cell responses including invasion, adhesion and proliferation have not yet been elucidated in detail. In this study, we report the regulatory roles and molecular mechanisms of PBSA in ovarian cancer cell fates including invasion and proliferation, and tumour‐derived angiogenic responses.

## 2 MATERIALS AND METHODS

### 2.1 Cell culture conditions

Human ovarian cancer cells (SKOV‐3) from American Type Culture Collection (ATCC) were grown in 10% foetal bovine serum‐Dulbecco's modified Eagle's medium (FBS‐DMEM) (Hyclone Laboratories). Human umbilical vein endothelial cells (HUVECs) were purchased from Lonza and used between passages 4 and 6 for all experiments. Cells were cultured in EGM‐2^®^ BulletKit media, according to the manufacturer's instructions (Lonza).

### 2.2 Reagents

**Figure 1:**
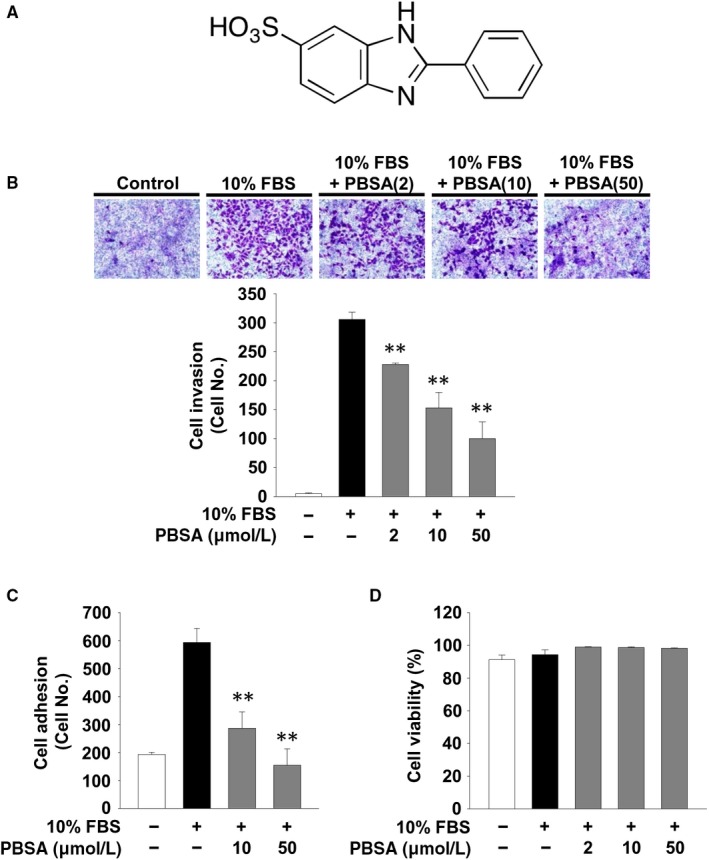
PBSA inhibits cell invasion and adhesion. A, The chemical structure of PBSA. B, Cell invasion, (C) adhesion and (D) viability assays were performed as described in Materials and methods. Quiescent SKOV‐3 cells were pre‐treated with PBSA for 30 min, followed by 10% FBS stimulation for 16 h (invasion), 2 h (adhesion) or 24 h (viability). Results from at least three independent experiments (mean ± SD) are presented as (B) the numbers of invasive cells, (C) the numbers of adherent cells or (D) the percentage of viable cells of total cell counts. Statistical significance is indicated (**P < .01, compared with 10% FBS‐treated cells)

2‐Phenylbenzimidazole‐5‐sulphonic acid (PBSA) was obtained from Sigma‐Aldrich. The structure of PBSA is presented in Figure 1A. The following chemical agents and antibodies were purchased from commercial sources: p38^MAPK^ inhibitor, SB203580 (Cayman Chemical); anti‐phospho‐Src (Y416), anti‐Src, anti‐phospho‐ERK (T202/Y204), anti‐phospho‐Akt (S473), anti‐phospho‐p70^S6K^ (T421/S424), anti‐phospho‐MKK3 (S189)/MKK‐6 (S207), anti‐MKK3, anti‐MKK6, anti‐phospho‐p38^MAPK^ (T180/Y182), anti‐phospho‐pRb (S780), anti‐phospho‐pRb (S807/S811), anti‐MMP‐2 and anti‐MMP‐9 (Cell Signaling); anti‐phospho‐FAK (Y397) and anti‐FAK (BD Biosciences); anti‐ERK, anti‐Akt, anti‐p70^S6K^, anti‐38^MAPK^, anti‐TIMP‐2, anti‐Cdk4, ant‐Cdk2, anti‐cyclin D, anti‐cyclin E, anti‐integrin β1, anti‐EGFR, anti‐FGFR‐1, anti‐VEGFR‐2, anti‐actin and mouse and rabbit IgG‐horseradish peroxidase conjugates (Santa Cruz Biotechnology Inc).

### 2.3 RNA purification and reverse transcriptase‐polymerase chain reaction (RT‐PCR)

Subconfluent SKOV‐3 cells in 100 mm dishes (1 × 10^6^ cells/well, SPL Life Sciences Co.) were serum‐starved for 24 hours in basal DMEM to synchronize cells in G_1_/G_0_ phase of the cell cycle and pre‐treated with PBSA (10, 50 μmol/L) for 30 minutes, followed by 10% FBS stimulation for 24 hours. In some experiments, quiescent cells were pre‐treated with PBSA or SB203580 for 30 minutes, followed by 10% FBS stimulation for 12 hours. After stimulation, cells were thoroughly rinsed with phosphate‐buffered saline (PBS, pH 7.4) to remove any residual PBSA or SB203580, and further incubated with 10% FBS for another 12 hours until the end of the 24 hours time‐point. Total RNA was purified with PureHelix^™^ RNA extraction solution (Nanohelix Co.). Integrity of RNA was checked by agarose gel electrophoresis and ethidium bromide staining. One microgram of RNA was used as template for each reverse transcriptase (RT)‐mediated polymerase chain reaction (PCR) using 1^st^ Strand cDNA Synthesis kit (BioAssay Co.). Primers for PCR were synthesized by Bioneer Corporation. Primer sequences were as follows: matrix metalloproteinases‐2 (MMP‐2), forward 5′‐GCTCAGATCCGTGGTGAGAT‐3′ and reverse 5′‐GGTGCTGGCTGAGTAGATCC‐3′; MMP‐9, forward 5′‐CAACATCACCTATTGGATCC‐3′ and reverse 5′‐TGGGTGTAGAGTCTCTCCCT‐3′; VEGF, forward 5′‐TCGGGCCTCCGAAACCATGA‐3′ and reverse 5′‐CCTGGTGAGAGATCTGGTTC‐3′; glyceraldehyde‐3‐phosphate dehydrogenase (GAPDH), forward 5′‐GAAGGTGAAGGTCGGAGTC‐3′ and reverse 5′‐GAAGATGGTGATGGGATTTC‐3′. Bands of interest were integrated and quantified by the use of NIH ImageJ version 1.51j8 software.

### 2.4 Western blot analysis

Quiescent SKOV‐3 cells in 6‐well plates (5 × 10^4^ cells/well, SPL Life Sciences Co.) were pre‐treated with PBSA for 30 minutes, followed by 10% FBS stimulation for different time‐points, as indicated. Cells were rinsed twice with ice‐cold PBS and lysed by incubation in 50 mmol/L Tris‐HCl (pH 7.4), 150 mmol/L NaCl, 1 mmol/L EDTA, 1% Triton X‐100, 10% glycerol, 0.5 μg/mL leupeptin, 1 μg/mL pepstatin A, 10 μg/mL aprotinin, 100 μg/mL AEBSF, 1 mmol/L sodium orthovanadate, 25 mmol/L sodium fluoride and 80 mmol/L β‐glycerophosphate for 30 minutes at 4ºC. Cell lysates were clarified at 12 500 *g* for 20 minutes at 4ºC, and the supernatants were subjected to Western blot analysis as described previously.^17^ All Western blots are representative of at least three independent experiments. Bands of interest were integrated and quantified by the use of NIH ImageJ version 1.51j8 software.

### 2.5 Cell viability and proliferation

Quiescent SKOV‐3 cells or HUVECs were pre‐treated with PBSA at different concentrations (2‐50 μmol/L) for 30 minutes in the presence or absence of SB203580 (5 μmol/L) as indicated, and further incubated with 10% FBS or EGM‐2^®^ BulletKit for 24 hours. In some experiments, quiescent SKOV‐3 cells were pre‐treated with PBSA for 30 minutes, followed by 10% FBS stimulation for 12 hours. After stimulation, cells were rinsed with PBS to remove any residual PBSA and further incubated with 10% FBS for another 12 hours. Cell viability was determined by a Muse^™^ cell analyser using cell count and viability assay kit (Merck Millipore), and the cell proliferation was quantified as previously described.^18^ The results from triplicate determinations (mean ± standard deviation) are presented as the percentage of viable cells of total cell counts or the fold increase of the untreated controls.

### 2.6 Cell cycle analysis

Quiescent SKOV‐3 cells were pre‐treated with PBSA (50 µmol/L) for 30 minutes and followed by 10% FBS stimulation for 24 hours. Cells were harvested with trypsin‐EDTA, rinsed with PBS and then fixed with ice‐cold 70% ethanol for at least 3 hours. After washing with PBS, cells were stained with Muse^™^ cell cycle reagent. The profile of cells in the G_1_/G_0_, S and G_2_/M phases of the cell cycle was analysed with a Muse^™^ cell analyser.

### 2.7 Cell adhesion assay

Subconfluent SKOV‐3 cells were detached with trypsin‐EDTA and allowed to recover in 10% FBS‐DMEM for 1 hour at 37ºC with gentle rocking. After recovery, cells were collected and resuspended in serum‐free DMEM. The cell suspension was pre‐treated with PBSA (10, 50 μmol/L) for 30 minutes, and followed by 10% FBS stimulation. Cells were plated on 96‐well plates (1.5 × 10^4^ cells/well) and further incubated for 2 hours at 37ºC. After incubation, unattached cells were removed by washing the wells three times with PBS. Attached cells were fixed with methanol and then stained with 0.04% Giemsa staining solution (Sigma‐Aldrich). Cells were photographed and counted. The results (mean ± standard deviation) are presented as the numbers of adherent cells.^19^

### 2.8 Cell invasion assay

The upper side of the transwell insert (Costar^Ⓡ^, 6.5 mm diameter insert, 8 μm pore size) (Corning Inc) was coated with 50 μL of 1 mg/mL Matrigel^Ⓡ^ (BD Biosciences) diluted in serum‐free DMEM. Aliquots (100 μL) of cells (6 × 10^5^ cells/mL) resuspended in serum‐free DMEM were added to the upper compartment of the Matrigel‐coated transwell, and 600 μL of serum‐free DMEM was added to the lower compartment. After culture for 2 hours, cells were pre‐treated with PBSA (2‐50 μmol/L) for 30 minutes in the presence or absence of SB203580 (5 μmol/L), followed by 10% FBS stimulation for 16 hours. The inserts were fixed with methanol, and using a cotton‐tipped swab, the non‐invasive cells were removed from the top of the membrane. After staining with 0.04% Giemsa staining solution, the numbers of invasive cells (mean ± standard deviation) were determined from six different fields using ×200 objective magnification.^20^

### 2.9 Zymogram analysis

Activities of MMPs were measured by zymography.^21^, ^22^ Aliquots of conditioned media collected from cells treated with PBSA (50 μmol/L) and 10% FBS for 16 hours were diluted in sample buffer and applied to 10% polyacrylamide gels containing 1 mg/mL gelatin (Sigma‐Aldrich) as a substrate. After electrophoresis, the gels were incubated in 2.5% Triton X‐100 for 1 hour to remove sodium dodecyl sulphate and allow re‐naturalization of MMPs and further incubated in developing buffer containing 50 mmol/L Tris‐HCl (pH 7.5), 150 mmol/L NaCl and 10 mmol/L CaCl_2_ for 16 hours at 37ºC. The gels were stained with 0.5% Coomassie brilliant blue R‐250 in 30% methanol‐10% acetic acid for 3 hours and followed by destaining with 30% methanol‐10% acetic acid. Gelatinolytic activities were detected as unstained bands against the background of the Coomassie blue‐stained gelatin. Bands of interest were integrated and quantified by the use of NIH ImageJ version 1.51j8 software.

### 2.10 Tube formation assay

Tube formation assay was performed to examine the ability of conditioned media from PBSA‐treated SKOV‐3 cells to modulate angiogenic responses in vitro. Quiescent cells were pre‐treated with PBSA (10, 50 µmol/L) for 30 minutes, followed by 10% FBS stimulation for 24 hours. In some experiments, quiescent cells were pre‐treated with PBSA or SB203580 for 30 minutes, followed by 10% FBS stimulation for 12 hours. Following stimulation, cells were washed with PBS to remove any residual PBSA or SB203580 and then further incubated with 10% FBS for another 12 hours. After stimulation, conditioned media were collected. Quiescent HUVECs (4 × 10^4^ cells/mL) were added to Matrigel^®^‐coated plates and treated with conditioned media for 12 hours. Tube formation was observed with an Olympus CKX41 inverted microscope (CAchN 10/0.25php objective) and ToupTek Toupview software (version ×86, 3.5.563, Hangzhou ToupTek Photonics Co.).^23^

### 2.11 VEGF enzyme‐linked immunosorbent assay (ELISA)

Quiescent SKOV‐3 cells were pre‐treated with PBSA (10, 50 µmol/L) for 30 minutes, followed by 10% FBS stimulation for 48 hours. In some experiments, quiescent cells were pre‐treated with PBSA or SB203580 for 30 minutes, followed by 10% FBS stimulation for 12 hours. Following stimulation, cells were washed with PBS to remove any residual PBSA or SB203580 and then further stimulated with 10% FBS for another 36 hours until the end of the 48 hours time‐point. After stimulation, conditioned media were collected. VEGF levels in the conditioned media were measured using VEGF ELISA kit (R&D Systems) and analysed at 450 nm with BioTek Synergy Mx microplate reader (BioTek Instruments).^24^

### 2.12 Rat aortic ring assay

Quiescent SKOV‐3 cells were pre‐treated with PBSA for 30 minutes, followed by 10% FBS stimulation for 12 hours. Following stimulation, cells were washed with PBS to remove any residual PBSA and then further stimulated with 10% FBS for another 12 hours until the end of the 24 hours time‐point. After stimulation, conditioned media were collected. Ex vivo angiogenic responses were quantified using rat aortic ring assay as described previously.^23^ Thoracic aorta was excised from 7‐ to 8‐week‐old male Sprague‐Dawley rats. Following careful removal of fat, tissue and branching vessels, the aortae were cut into 1 mm long cross‐sections, placed on Matrigel‐coated plates, covered with an additional layer of Matrigel. The rings embedded in Matrigel were incubated with conditioned media for 3 days and then incubated with fresh conditioned media every other day and photographed on the 7^th^ day using ×40 objective magnification. The area of angiogenic sprouting was quantified using Adobe PhotoShop software. The animal studies were performed in accordance with the institutional guidelines. The experimental protocol had received prior approval from the Institutional Animal Care and Use Committee at Dankook University.

### 2.13 Statistical analysis

Statistical analysis was performed using Student's *t* test and was based on at least three different experiments. The results were considered to be statistically significant when *P* < .05.

## 3 RESULTS

### 3.1 PBSA inhibits cell invasion through the regulation of MMP expression and activity

**Figure 2:**
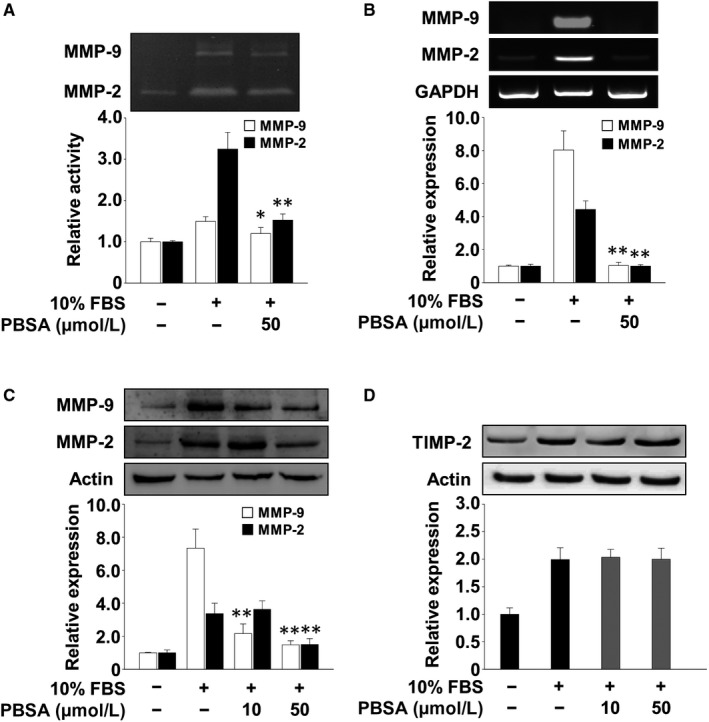
PBSA suppresses MMP expression and activity. Quiescent SKOV‐3 cells were pre‐treated with PBSA for 30 min, followed by 10% FBS stimulation for (A) 16 h or (B, C and D) 24 h. A, Gelatin zymogram analysis was carried out using conditioned media from cell culture treated as described above. Zymogram gel loading was normalized to total protein concentration. The levels of MMP‐2, MMP‐9 and TIMP‐2 were determined by (B) RT‐PCR and (C and D) Western blot analyses. Results shown are representative of at least three independent experiments. Integrated density values were normalized to untreated controls. Statistical significance is indicated (*P < .05, **P < .01, compared with 10% FBS‐treated cells)

Cell invasion, migration and adhesion are closely associated with cancer progression and aggressiveness.^6^ We first examined the ability of PBSA to regulate cell invasion and adhesion in p53‐deficient SKOV‐3 ovarian cancer cells. PBSA treatment dose‐dependently inhibited mitogen‐induced cell invasion and adhesion (Figure 1B,C) and did not alter cell viability (Figure 1D), indicating the potential of PBSA in the regulation of cell invasion and adhesion without cytotoxicity. In addition, our initial experiments demonstrated that PBSA significantly inhibited the invasion of p53 wild‐type ovarian cancer cells including A2780, OVTOKO and TOV‐21G to levels similar to that observed in SKOV‐3 cells (data not shown). We next examined the changes in expression levels and activities of MMPs, which play important roles in cell invasion, migration, adhesion and proliferation by degrading extracellular matrix (ECM) components and cell surface proteins.^6^, ^7^ The levels of MMP‐2 activity in the conditioned media from cell cultures were shown to be higher than those of MMP‐9 (Figure 2A), consistent with previous findings.^4^ PBSA treatment significantly reduced the proteolytic activities of MMP‐2 and MMP‐9 in mitogen‐stimulated SKOV‐3 cells (Figure 2A), and this inhibitory effect of PBSA was found to be mediated through the down‐regulation of MMP‐2 and MMP‐9 expression (Figure 2B,C). In contrast, PBSA treatment did not alter the levels of tissue inhibitor of metalloproteinases‐2 (TIMP‐2), an endogenous inhibitor of MMPs (Figure 2D). Although it cannot be absolutely excluded that PBSA‐mediated inhibition of cell invasion is partly mediated by regulation of other MMPs or TIMPs not investigated in this study, these findings suggest that PBSA‐mediated down‐regulation of MMP expression and proteolytic activity, in particular MMP‐2 and MMP‐9, might inhibit the invasive potential of ovarian cancer cells in response to mitogenic stimulation.

### 3.2 PBSA inhibits cell proliferation through the regulation of cell cycle‐related proteins

**Figure 3:**
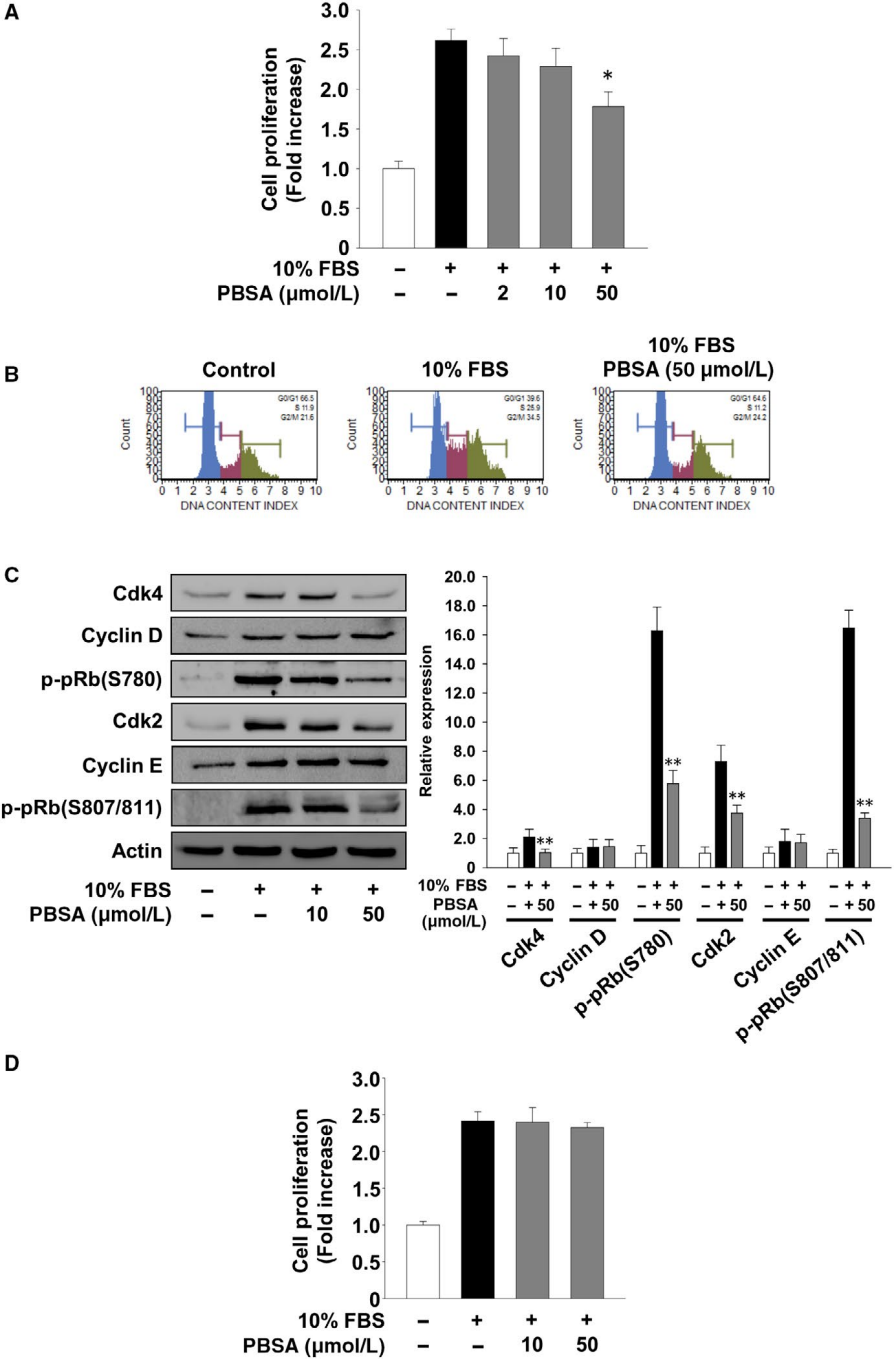
PBSA inhibits cell proliferation by down‐regulating cell cycle‐related proteins. Quiescent SKOV‐3 cells were pre‐treated with PBSA for 30 min, followed by 10% FBS stimulation for 24 h. A, Cell proliferation results from at least three independent experiments (mean ± SD) are presented as the fold increase of the untreated controls. B, Cell cycle and (C) Western blot analyses were performed as described in Materials and methods. Results shown are representative of at least three independent experiments. Statistical significance is indicated (*P < .05, **P < .01, compared with 10% FBS‐treated cells). D, Quiescent SKOV‐3 cells were pre‐treated with PBSA for 30 min, followed by 10% FBS stimulation for 12 h. Following stimulation, cells were rinsed with PBS to remove any residual PBSA and further incubated with 10% FBS for another 12 h until the end of the 24 h time‐point. Results from at least three independent experiments are presented as the fold increase of the untreated controls

We next examined the effect of PBSA on proliferation and cell cycle progression in SKOV‐3 cells. PBSA treatment dose‐dependently inhibited mitogen‐stimulated cell proliferation (Figure 3A), similar to the patterns of other ovarian cancer A2780 and OVTOKO cells (data not shown). As shown in Figure 3B, mitogenic stimulation increased the percentage of cells in S phase (11.9% vs 25.9%) and G_2_/M phase (21.6% vs 34.5%) and concomitantly decreased that in G_1_ phase (66.5% vs 39.6%), as compared with untreated controls. However, PBSA treatment blocked mitogen‐induced changes in the cell cycle phase distribution to the levels observed in the untreated controls. These data suggest that PBSA inhibits mitogen‐induced G_1_/S phase transition, resulting in G_1_ arrest. Based on these findings, we next analysed the changes of cell cycle‐related proteins in PBSA‐treated cells. PBSA treatment markedly suppressed the expression of cell cycle‐dependent kinases (Cdks) such as Cdk4 and Cdk2, but not cyclins, resulting in pRb hypophosphorylation (Figure 3C). These findings are well correlated with inhibition of cell cycle progression and proliferation (Figure 3A,B). In addition, this anti‐proliferative activity of PBSA was abrogated after the withdrawal of PBSA at the 12 hours time‐point, suggesting that anti‐proliferative activity of PBSA may be reversible (Figure 3D). Collectively, these findings indicate that PBSA possesses anti‐invasive, anti‐adhesive and anti‐proliferative activity against ovarian cancer SKOV‐3 cells.

### 3.3 PBSA inhibits integrin β1 expression and p38^MAPK^ phosphorylation

**Figure 4:**
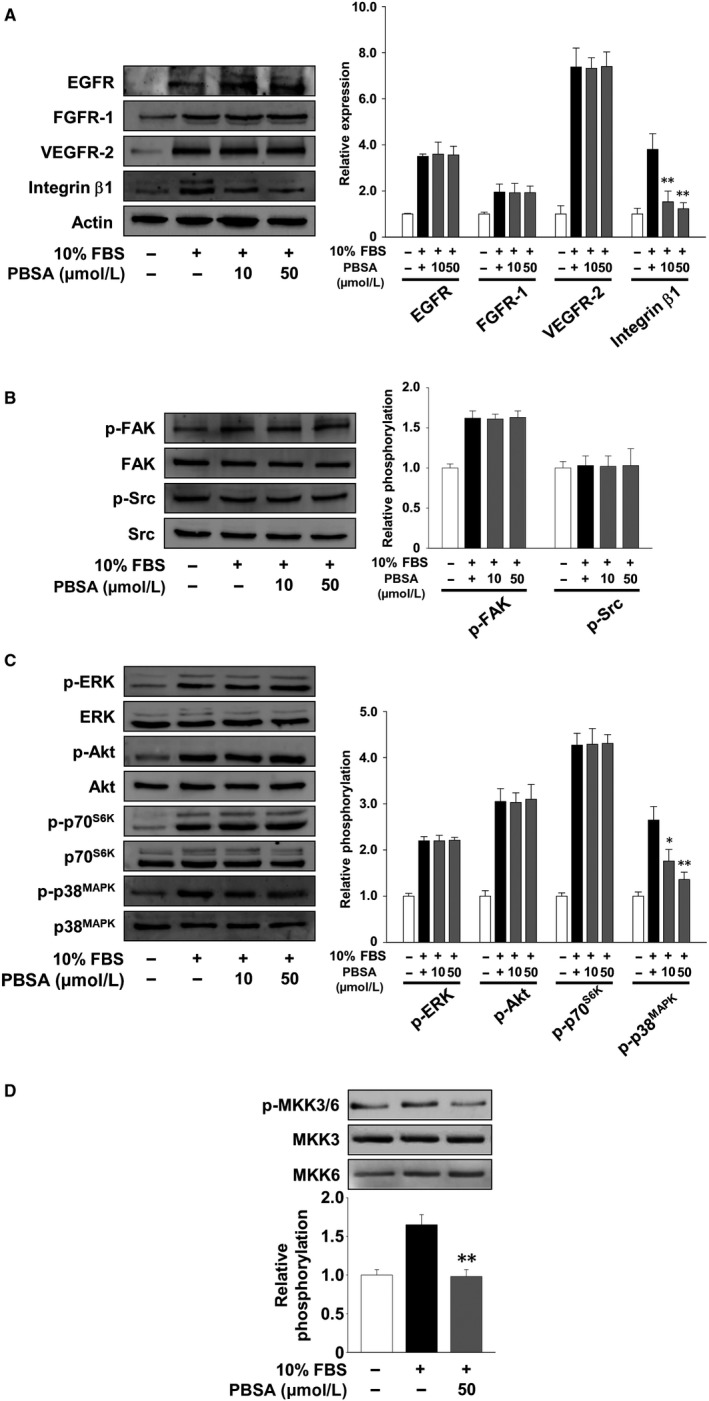
PBSA inhibits integrin β1 expression and MKK3/6‐p38^MAPK^ activation. Quiescent SKOV‐3 cells were pre‐treated with PBSA for 30 min, followed by 10% FBS stimulation for (A) 24 h, (B) 5 min, (C) 15 min or (D) 10 min. Results shown are representative of at least three independent experiments. Integrated density values were normalized to untreated controls. Statistical significance is indicated (*P < .05, **P < .01, compared with 10% FBS‐treated cells)

To investigate the molecular mechanisms and targets of PBSA in regulating cell invasion and proliferation, we analysed the changes in the expression of cell surface receptor proteins such as vascular endothelial growth factor receptor‐2 (VEGFR‐2), fibroblast growth factor receptor‐1 (FGFR‐1), epidermal growth factor receptor (EGFR) and integrin β1, and activation of their downstream signalling pathways including focal adhesion kinase (FAK), Src kinase (Src), extracellular signal‐regulated kinase (ERK), Akt, p70 S6 kinase (p70^S6K^) and p38 mitogen‐activated protein kinase (p38^MAPK^) in PBSA‐treated cells. As shown in Figure 4A, PBSA treatment markedly inhibited mitogen‐induced expression of integrin β1, which plays important roles in cell adhesion, migration and invasion associated with cancer growth and progression.^5^, ^25^, ^26^ However, PBSA did not modulate the expression of receptor tyrosine kinases (RTKs) including VEGFR‐2, FGFR‐1 and EGFR, raising the possibility that anti‐cancer activity of PBSA might be mediated by the regulation of downstream signalling components of RTKs and integrins. PBSA treatment specifically inhibited the phosphorylation of p38^MAPK^, but not that of FAK, Src, ERK, Akt and p70^S6K^, in mitogen‐stimulated SKOV‐3 cells (Figure 4B,C). Therefore, we next examined the effect of PBSA on the phosphorylation of mitogen‐activated protein kinase kinase 3 (MKK3) and MKK6, direct upstream protein kinases of p38^MAPK^.^27^ As shown in Figure 4D, PBSA markedly inhibited mitogen‐induced MKK3/6 phosphorylation to the levels observed in the untreated controls. These data suggest that anti‐invasive and anti‐proliferative activities of PBSA against ovarian cancer cells are mediated, at least in part, through the inhibition of integrin β1 expression and inactivation of MKK3/6‐p38^MAPK^‐dependent signalling pathway.

### 3.4 Anti‐cancer activities of PBSA are mediated by the inactivation of p38^MAPK^‐dependent signalling pathway

**Figure 5:**
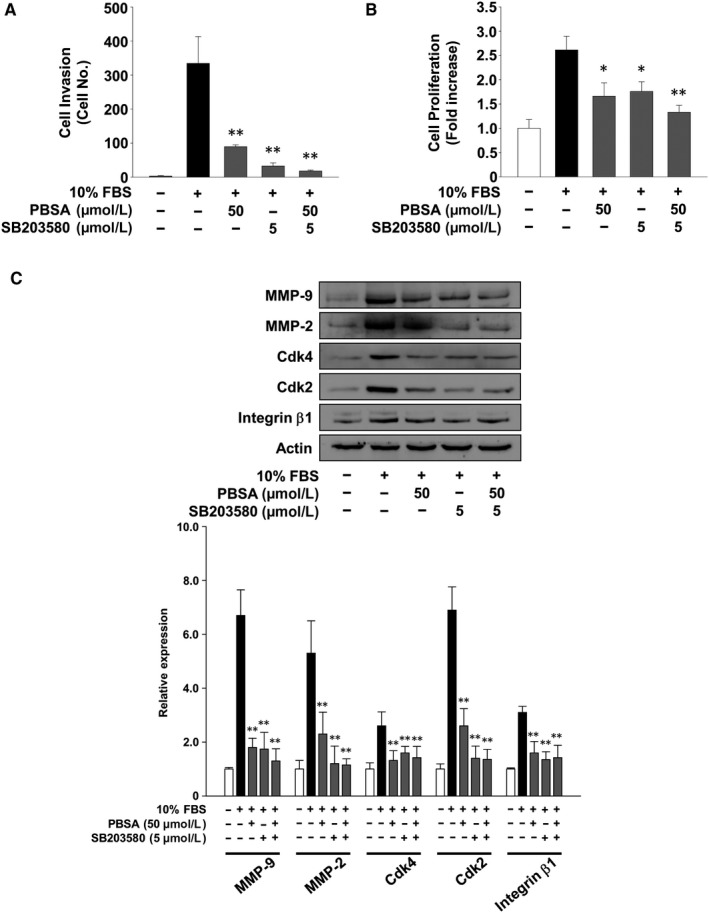
PBSA inhibits cell invasion and proliferation through the inactivation of p38^MAPK^. Quiescent SKOV‐3 cells were pre‐treated with PBSA for 30 min in the presence or absence of SB203580, followed by 10% FBS stimulation for (A) 16 h or (B and C) 24 h. Results from at least three independent experiments (mean ± SD) are presented as (A) the numbers of invasive cells or (B) the fold increase of the untreated controls. C, Results shown are representative of at least three independent experiments. Integrated density values were normalized to untreated controls. Statistical significance is indicated (*P < .05, **P < .01, compared with 10% FBS‐treated cells)

To directly examine the contribution of inactivation of p38^MAPK^ to the anti‐cancer activity of PBSA, we investigated the changes in cell invasion and proliferation in the presence or absence of SB203580, an inhibitor of p38^MAPK^ pathway, in mitogen‐stimulated SKOV‐3 cells. SB203580 treatment mimicked the inhibitory effect of PBSA on mitogen‐stimulated cell invasion and proliferation (Figure 5A,B). SB203580 treatment also suppressed mitogen‐induced expression of MMP‐9, MMP‐2, Cdk4, Cdk2 and integrin β1 (Figure 5C). These data are consistent with previous cell invasion and cell cycle‐related protein expression experiments (Figures 2C, 3C and 4A). The addition of SB203580 did not significantly enhance the ability of PBSA to inhibit cell invasion and proliferation, suggesting that PBSA and SB203580 may share similar mechanisms of action in the regulation of cellular responses. Collectively, these findings demonstrate that inactivation of p38^MAPK^ mediates anti‐invasive and anti‐proliferative activities of PBSA against ovarian cancer SKOV‐3 cells.

### 3.5 PBSA inhibits endothelial cell tube formation in vitro and angiogenic sprouting ex vivo through p38^MAPK^‐dependent regulation of VEGF expression and secretion in ovarian cancer cells

**Figure 6:**
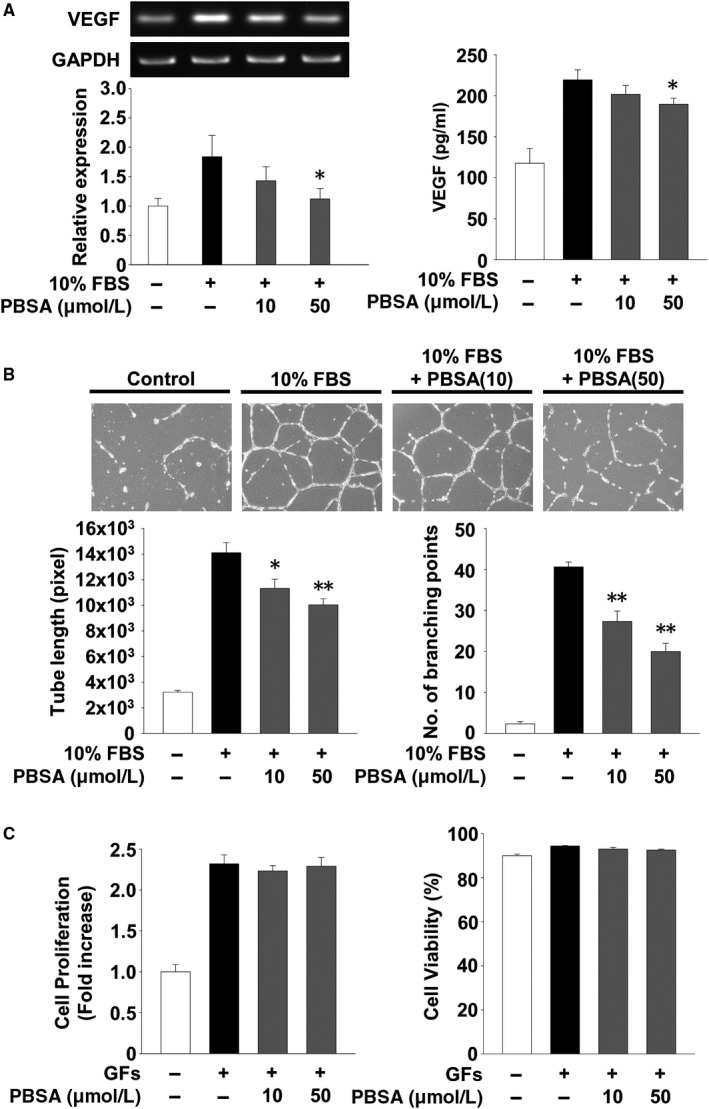
PBSA inhibits endothelial tube formation through the down‐regulation of VEGF. A, Quiescent SKOV‐3 cells were pre‐treated with PBSA for 30 min, followed by 10% FBS stimulation for (left panel) 24 h or (right panel) 48 h. RT‐PCR and ELISA analyses were performed as described in Materials and methods. B, HUVEC tube formation assay was performed using conditioned media from SKOV‐3 cell culture treated as described above (A, left panel). Values represent the mean ± SD of at least three independent experiments. Statistical significance is indicated (*P < .05, **P < .01, compared with 10% FBS‐treated cells). C, Quiescent HUVECs were incubated for 24 h in complete media containing growth factors with or without PBSA. Results from at least three independent experiments are presented as the fold increase of the untreated controls (left panel) or the percentage of viable cells of total cell counts (right panel)

**Figure 7:**
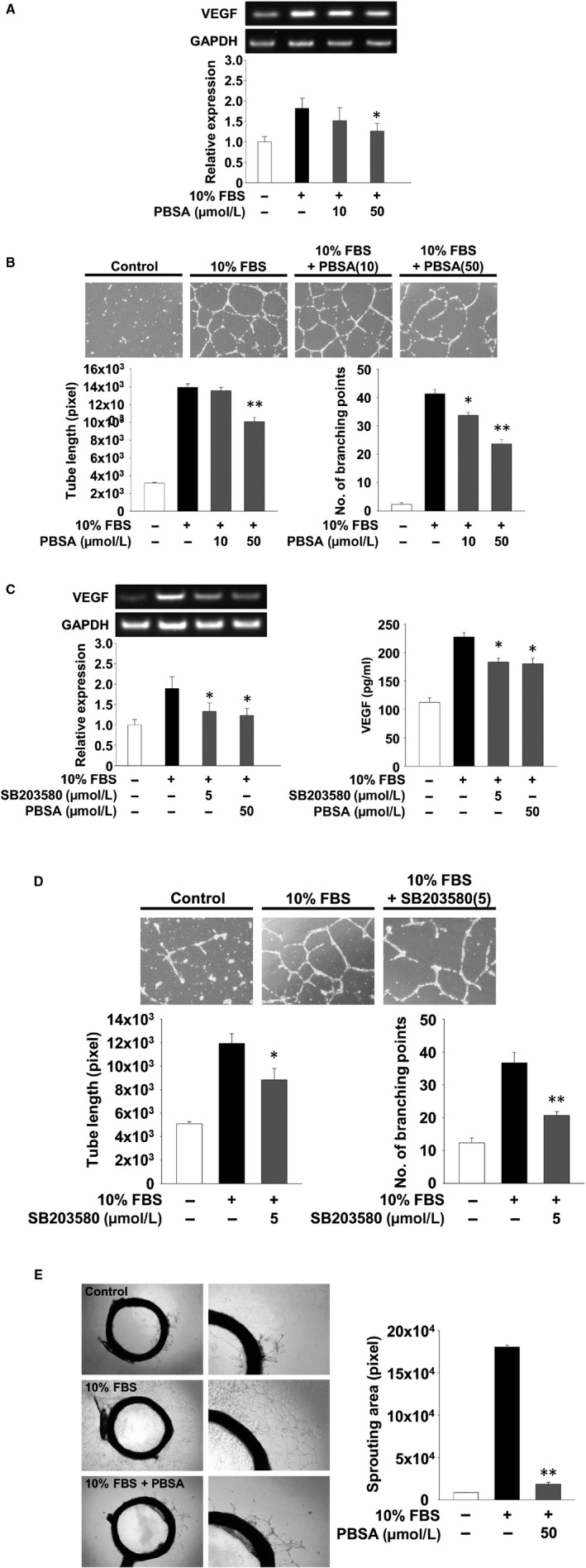
PBSA inhibits angiogenic responses by p38^MAPK^‐dependent down‐regulation of VEGF. Quiescent SKOV‐3 cells were pre‐treated with PBSA or SB203580 for 30 min, followed by 10% FBS stimulation for 12 h. After stimulation, cells were washed with PBS to remove any residual PBSA or SB203580 and further incubated with 10% FBS for another 12 h or (C, right panel) 36 h. (A and C, left panel) RT‐PCR was performed as described in Materials and methods. B and D, tube formation, (C, right panel) ELISA and (E) rat aortic ring assays were performed using conditioned media from SKOV‐3 cell culture treated as described above. Values represent the mean ± SD of at least three independent experiments. Statistical significance is indicated (*P < .05, **P < .01, compared with 10% FBS‐treated cells)

In the tumour microenvironment, cancer cells produce and secrete numerous biologically active molecules such as growth factors and cytokines, which are closely associated with angiogenic and metastatic responses.^28^, ^29^, ^30^ We analysed the changes in the expression and secretion of a key angiogenic factor VEGF in PBSA‐treated SKOV‐3 cells. As shown in Figure 6A, PBSA treatment significantly suppressed mitogen‐induced expression and secretion of VEGF, indicating that PBSA‐mediated inhibition of VEGF secretion may be due in part to down‐regulation of VEGF mRNA expression. To determine whether the biologically active molecules including VEGF secreted from SKOV‐3 cells affect the responses of other neighbouring cells, we performed in vitro angiogenesis assay using conditioned media from SKOV‐3 cells in the presence or absence of PBSA (Figure 6B). The conditioned media from PBSA‐treated SKOV‐3 cells markedly prevented mitogen‐induced capillary‐like structure formation of HUVECs. In contrast, PBSA did not directly affect mitogen‐induced proliferation or viability in HUVECs, indicating that in vitro anti‐angiogenic activities of PBSA are not mediated through the regulation of endothelial cell proliferation or viability (Figure 6C). In addition, the suppressive effect of PBSA‐treated cell conditioned media on in vitro angiogenic responses appeared to be irreversible until the end of this experiment, since the withdrawal of PBSA at the 12 hours time‐point did not significantly alter PBSA‐mediated suppression of VEGF expression and endothelial cell tube formation (Figure 7A,B), compared with previous findings (Figure 6A,B). Although the types and levels of secreted molecules in PBSA‐treated SKOV‐3 cells remain to be further elucidated, these findings suggest that PBSA‐mediated suppression of VEGF expression and secretion in SKOV‐3 cells may be one of the major factors in regulating tumour‐derived angiogenesis. To investigate whether PBSA‐mediated suppression of VEGF expression and subsequent in vitro angiogenesis is mediated by the inactivation of p38^MAPK^, we performed RT‐PCR and ELISA analyses for VEGF, and endothelial cell tube formation assay using SB203580‐treated SKOV‐3 cells or conditioned media. SB203580 treatment markedly inhibited expression and secretion of VEGF to similar levels as observed with PBSA treatment (Figure 7A,C). In addition, the conditioned media from SB203580‐treated SKOV‐3 cells mimicked the PBSA‐mediated inhibition of endothelial tube formation (Figure 7B,D). Finally, the conditioned media from PBSA‐treated SKOV‐3 cells markedly inhibited microvessel outgrowth in the rat aortic ring assay (Figure 7E). Taken together, these results convincingly demonstrate the pharmacological properties of PBSA in regulating ovarian cancer cell responses in vitro, and capillary‐like tubular structures in vitro and angiogenic sprouting ex vivo through the selective inactivation of p38^MAPK^‐dependent signalling pathway.

## 4 DISCUSSION

2‐phenylbenzimidazole‐5‐sulphonic acid has widely been used as a UV‐B filter in sunscreen formulations and cosmetics. Although some potential adverse effects and risk assessments of PBSA to natural environment and human health have been conducted, the pharmacological effects and molecular mechanisms of PBSA in cancer growth and progression as well as angiogenesis have not yet been investigated.^12^, ^13^, ^14^, ^31^

Integrins are transmembrane receptors that modulate cell‐ECM and cell‐cell interactions and play pivotal roles in various pathological conditions including cancer and normal cell/tissue responses. Integrin‐mediated cellular responses are complicatedly regulated by interactions with extracellular ligands, intracellular molecules or cell surface molecules including RTKs.^32^, ^33^, ^34^, ^35^, ^36^ These complex interactions may partially explain the limitation of integrin‐ or RTK‐targeted therapeutics in clinical trials and applications. Therefore, a detailed understanding of the molecular mechanisms and subsequent identification of key targets in integrin/RTK signalling pathways are prerequisite for the development of highly effective therapeutic approaches and agents.

The p38^MAPK^‐dependent signalling pathway is closely involved in inflammation and cancer development as well as tissue homoeostasis.^37^ Identification of the key roles and regulatory mechanisms of p38^MAPK^ in a number of physiological and pathological processes may provide therapeutic perspectives and strategies for preclinical and clinical applications.^38^, ^39^ Recent studies demonstrate that activation and expression of p38^MAPK^ play important roles in regulating ovarian cancer cell responses including invasion and proliferation.^40^, ^41^ Thus, it is important to note that selective blockade of p38^MAPK^ activation and expression provides key information for the treatment of ovarian cancer.

In this study, we demonstrate that PBSA negatively regulates p53‐deficient ovarian cancer cell fates including invasion, adhesion and proliferation, and angiogenic responses such as capillary‐like structure formation and microvessel outgrowth without affecting endothelial cell proliferation or cytotoxicity. The mechanism of these anti‐cancer and anti‐angiogenic activities of PBSA involves inactivation of MKK3/6‐p38^MAPK^‐dependent signalling pathways and subsequent down‐regulation of MMP‐9, MMP‐2, Cdk4, Cdk2, integrin β1 and VEGF expression, which are closely associated with cancer cell growth and progression as well as angiogenesis. In addition, PBSA inhibits the invasion and proliferation of p53 wild‐type ovarian cancer cells (data not shown). These observations demonstrate that anti‐cancer activity of PBSA against ovarian cancer is independent of p53 expression. Although functions and targets of PBSA within the tumour microenvironment, which includes cancer cells, immune cells, fibroblasts, secreted proteins, ECM components and blood vessels, remain to be further elucidated, our initial experiments show that PBSA inhibits proliferation of p53 wild‐type human breast cancer and p53‐deficient non–small‐cell lung cancer cells (data not shown). These data may suggest the broad spectrum therapeutic potential of PBSA for the treatment of cancer, independently of cell/tissue types or p53 tumour suppressor gene status. Finally, this study describes for the first time the pharmacological roles and molecular targets of PBSA in the regulation of ovarian cancer cell fates and tumour‐derived angiogenesis and warrants further in‐depth evaluation of PBSA for the development of anti‐ cancer agents.

## CONFLICT OF INTEREST

The authors confirm that there are no conflicts of interest.

## AUTHORS’ CONTRIBUTIONS

MSK, JHK, KBK and DWS designed and performed research and wrote the manuscript; EKA, YRC, SH and CHL performed research; GUB and JSO analysed data; KBK and DWS were responsible for supervising the entire project and writing the manuscript.

## Data Availability

The data that support the findings of this study are available from the corresponding author upon reasonable request.
